# The Impact of Bending Stress on the Performance of Giant Magneto-Impedance (GMI) Magnetic Sensors

**DOI:** 10.3390/s17030640

**Published:** 2017-03-20

**Authors:** Julie Nabias, Aktham Asfour, Jean-Paul Yonnet

**Affiliations:** Université Grenoble Alpes, CNRS, Grenoble INP (Institute of Engineering Université Grenoble Alpes), G2Elab, F-38000 Grenoble, France; Julie.Nabias@g2elab.grenoble-inp.fr (J.N.); Jean-Paul.Yonnet@g2elab.grenoble-inp.fr (J.-P.Y.)

**Keywords:** Giant Magneto-Impedance (GMI), amorphous wire, bending stress, flexible

## Abstract

The flexibility of amorphous Giant Magneto-Impedance (GMI) micro wires makes them easy to use in several magnetic field sensing applications, such as electrical current sensing, where they need to be deformed in order to be aligned with the measured field. The present paper deals with the bending impact, as a parameter of influence of the sensor, on the GMI effect in 100 µm Co-rich amorphous wires. Changes in the values of key parameters associated with the GMI effect have been investigated under bending stress. These parameters included the GMI ratio, the intrinsic sensitivity, and the offset at a given bias field. The experimental results have shown that bending the wire resulted in a reduction of GMI ratio and sensitivity. The bending also induced a net change in the offset for the considered bending curvature and the set of used excitation parameters (1 MHz, 1 mA). Furthermore, the field of the maximum impedance, which is generally related to the anisotropy field of the wire, was increased. The reversibility and the repeatability of the bending effect were also evaluated by applying repetitive bending stresses. The observations have actually shown that the behavior of the wire under the bending stress was roughly reversible and repetitive.

## 1. Introduction

The use of Giant Magneto-Impedance (GMI) wires as sensing elements for electrical current sensors in real industrial environments raises important issues related to the parameters that influence the sensor. These parameters can dramatically affect the accuracy and reliability of the measurement. They may include—but are not limited to—the temperature and the surrounding magnetic fields produced, for example, by other conductors in close proximity to the conductor of interest.

Mechanical effects can also be encountered in a number of structures of GMI-based current sensors. In fact, in some situations, the GMI wire has to be deformed since it needs to be aligned with the magnetic field produced by the conductor carrying the measured electrical current. Indeed, this is typically the case in some prototypes of current sensors where the sensor has a toroidal structure [1,2,3,4,5,6,7], unlike other prototypes that do not involve deformation [8,9].

In the case of toroidal configuration, some studies have already been conducted to evaluate the impact of the deformation of the amorphous wire on the GMI effect [1].

The effects of tensile [10,11,12,13,14,15,16,17] and torsional [18,19] stresses have been intensively investigated in amorphous microwires with low (to vanishing or slightly negative) magnetostriction. Stresses induced during certain fabrication processes, such as cold drawing, can also affect the domain structure of the wires and influence the GMI ratio and field sensitivity [20].

By far, the bending stress effect in GMI amorphous wires is actually less known. Nevertheless, the investigation of this effect is particularly important in applications such as current sensors. In fact, it is important to ensure that the potential variations of the intrinsic relevant quantities of the GMI curve due to bending stress will not affect the final response of the sensor. Or at least, the knowledge of the impact of the bending on these relevant quantities has to be developed so as to allow for adequate solutions to minimizing this impact.

In a sensor application, the relevant quantities to be considered include the offset resulting from the field biasing, the intrinsic sensitivity at the bias point, and the GMI ratio.

**Figure 1:**
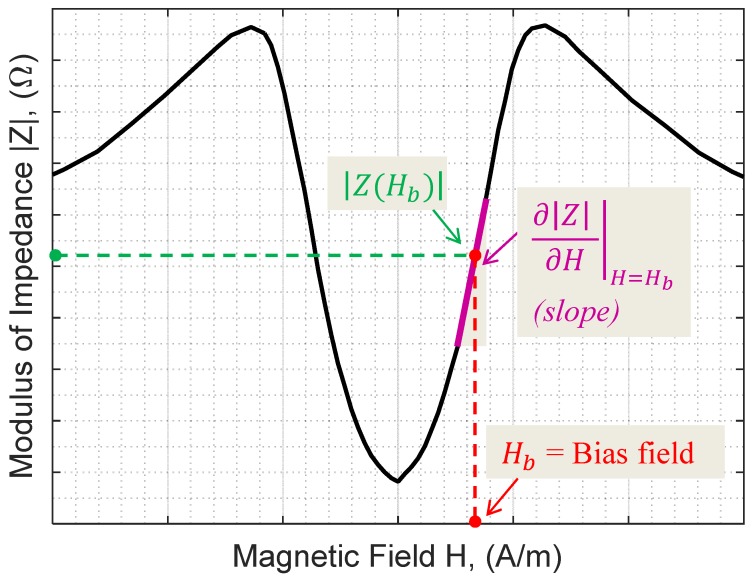
A typical Giant Magneto-Impedance (GMI) curve, |Z(H)|.

These quantities are illustrated in Figure 1, which presents typical nonlinear GMI characteristics (modulus of the impedance, |Z(H)|, as a function of the magnetic field H) for an amorphous wire.

As is well-known, in the classical use of a GMI wire for developing a linear sensor, the wire must be biased by a magnetic field, $H_{b}$, in the region that exhibits near-linear behavior. This field biasing gives rise to an offset voltage at the final sensor output when the measured magnetic field is zero, since the output voltage is directly proportional to the value of the impedance at the bias field ($\left|Z\left(H_{b}\right)\right|$ in Figure 1). Electronic canceling of the offset voltage is usually employed. However, this canceling should be effective only if the value of $\left|Z\left(H_{b}\right)\right|$ does not change under the parameters of influence.

In addition to linearity considerations, the bias point is also chosen to obtain a maximum sensitivity. At the bias point, the intrinsic sensitivity of the sensor is defined by

(1) $${S\left(H_{b}\right)=\frac{\partial \left|Z\right|}{\partial H}|}_{H=H_{b}}.$$

This quantity ${\frac{\partial \left|Z\right|}{\partial H}|}_{H=H_{b}}$ will dramatically determine the final sensitivity of the sensor in open-loop operation and, if it is not high enough, it could also impact the sensitivity in closed-loop operation.

A third quantity to be considered is the GMI ratio, ∆*Z*/*Z*, defined as

(2) $$\frac{∆Z}{Z}\;\left(\%\right)=\frac{\left|⏐Z\left(H\right)⏐-⏐Z\left(H_{max}\right)⏐\right|}{\left|Z\left(H_{max}\right)\right|}\times 100$$

where a commonly used value for $H_{max}$ is the saturation field of the magnetic material. In practice, $H_{max}$ is generally the maximum field available in a given setup. The GMI ratio obviously contributes to fixing the total dynamic range of the open-loop sensor. In fact, a higher GMI ratio allows more excursion of the measured field around the bias point.

Under the parameters of influence, the offset, the intrinsic sensitivity, *S*, and the GMI ratio are potentially subject to changes that can largely degrade the sensor’s performance and reliability. According to the requirements of each application, one or several of these quantities have to be optimized.

In this paper, an experimental study is conducted to evaluate the impact of the bending stress, as an influence parameter, on the offset, the intrinsic sensitivity, and the GMI ratio of an amorphous wire. A full description of the measurement setup and experimental conditions are given. The first obtained results are illustrated and discussed. Tests of the repeatability and reversibility of the bending stress effect are also included.

## 2. Materials and Methods

**Figure 2:**
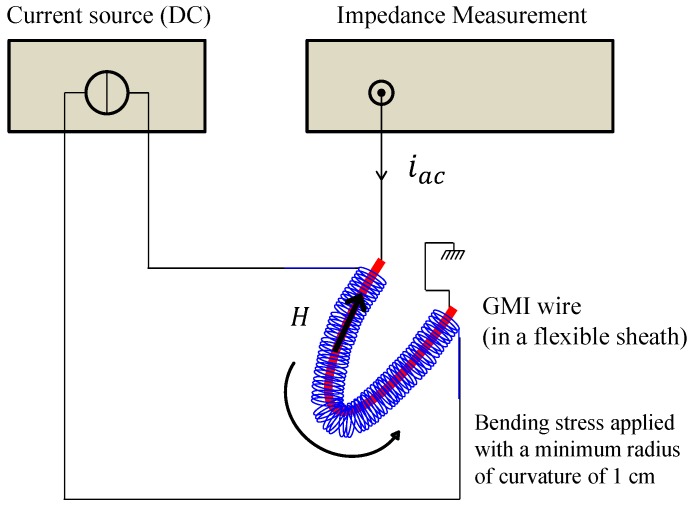
The experimental setup. The GMI wire was 90 mm in length and excited by an AC current with a 1 mA amplitude and a 1 MHz frequency. In this figure, the wire is in a bent position.

Figure 2 shows the developed experimental setup. The used amorphous Co-rich wires (Co-Fe-Si-B) with a 100 µm diameter were from Unitika Ltd., obtained with the in-rotating-water spinning process. No additional treatments were performed on these wires.

For all experiments, a wire 90 mm long was cut and placed inside a flexible envelope (sheath). The extremities of the wire were soldered to copper wires for electrical connections and measurement purposes. The external magnetic field, H, was applied to the wire using a 270-turn coil placed around the flexible sheath. The maximum available field $H_{max}$ was 1 kA/m.

In all the experiments, the impedance was measured when the GMI wire was supplied by a high frequency (HF) current, $i_{ac}$, with a 1 MHz frequency and a 1 mA amplitude. These values were only justified by the requirements of our application, where the sensor’s energy consumption and the simplicity of implementation of the conditioning electronics are issues. Additionally, with relatively low AC currents, the nonlinear effects of the GMI (nonlinearity between the current and the voltage across the wire) were avoided [21].

The bending stress was simultaneously applied to the GMI wire, sheath, and coil. The minimum measured a radius of curvature, which is representative of the applied strain, was about 10 mm. This yields a calculated stress of approximately 800 MPa in tension and compression along the wire.

## 3. Results and Discussion

**Figure 3:**
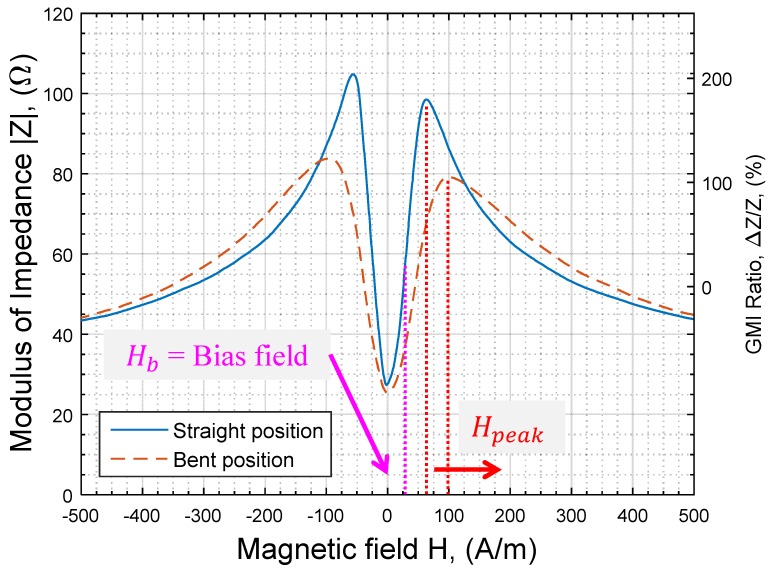
Change of |Z(H)| and ∆*Z*/*Z* with the bending stress.

Figure 3 shows the change of |Z(H)| and ∆*Z*/*Z* with the applied bending stress.

One could note that the |Z(H)| curves of Figure 3 are slightly asymmetric, in both straight and bent positions. This intrinsic asymmetry, which has been intensively investigated in the literature, could have several causes [22]. While the study of the asymmetry is well beyond the scope of this paper, it seems that its origin is related to the hysteresis of the GMI material. The curves of Figure 3 have actually been plotted in the case of an increasing magnetic field (the asymmetry is reversed for a decreasing field). The asymmetry does not, however, withdraw the validity and the generality of the obtained results.

### 3.1. GMI Ratio and Anisotropy Field

For simplicity of illustration and explanation, we are interested, without a loss of generality, in the positive fields region only.

When the used sample was not bent (straight position), a typical maximum GMI ratio, ${\left(∆Z/Z\right)}_{max}$, of more than 220% was obtained. The field of the maximum impedance and the GMI ratio, $H_{peak}$, which is related to the anisotropy field $H_{k}$, was nearly equal to 64 A/m.

In the bent position, a decrease in the ratio ${\left(∆Z/Z\right)}_{max}$ to less than 160% was observed with $H_{peak}$, increasing to more than 100 A/m.

This result seems to be in agreement with the one that could be obtained with a tensile stress [10,11,12,13,14,15,16,17]. The used amorphous wires have a nearly zero magnetostriction [23]. Nevertheless, the domain structure of these wires is often considered to be similar to that of negative magnetostrictive wires [22]. In such a case, the quenched-in stresses due to fabrication by rapid solidification may cause the surface anisotropy to be circular and the inner anisotropy to be perpendicular to the wire axis, thereby leading to the formation of a specific domain structure, which consists of outer shell circular domains and inner core roughly axial domains [22]. This means that the anisotropy is circumferential in the outer shell of the wire.

**Figure 4:**
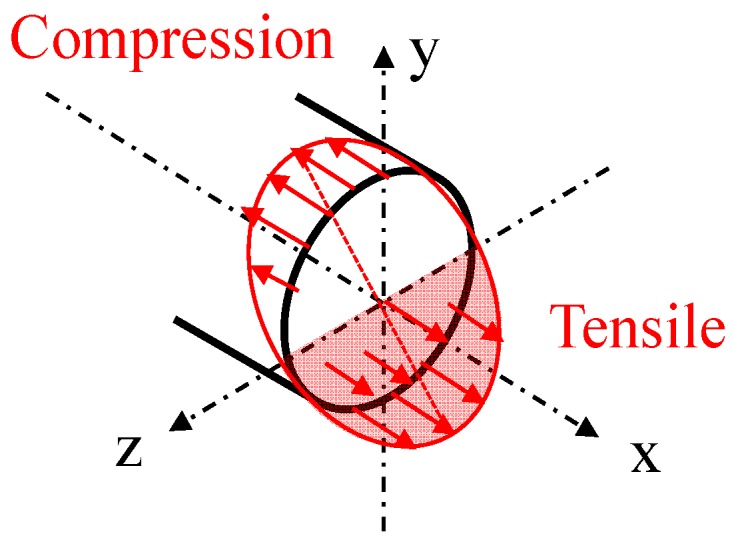
Representation of a bending stress.

In a first approximation, a bending stress could be assumed to be a combination of tensile stress along the outer fiber and compressive stress along the inner fiber of the wire, as illustrated by Figure 4. There is no deformation of the fiber along the neutral axis.

In fact, the amplitude of the strain, *σ*, applied on the section of the wire can be calculated using the following formula:

(3) $$\sigma =-E\times \frac{y}{\rho }$$

where *E* is the Young’s modulus of the wire (16,000 kg/mm^2^ in our case), *y* is the distance of a given fiber to the neutral axis, and *ρ* is the radius of curvature.

According to this formula, *σ* is positive when the distance *y* is counted negatively (outer fibers of the wire), and *σ* is negative when the distance *y* is counted positively (inner fibers of the wire). Therefore, the strain should theoretically be compensated in the total volume of the wire, as there is the exact same amount of tensile and compressive stress along the wire. However, this simplified model should be considered cautiously, as it appears from our experiments that there is a change in |Z(H)| with the applied bending stress. This would actually suggest that the effect of the compressive stress is not exactly opposed to the effect of the tensile stress. It seems that the two effects are mixed in some manner, resulting in a dominating tensile stress effect.

The effect of the tensile stress has been intensively studied [10,11,12,13,14,15,16,17]. For a negative magnetostrictive wire, a tensile stress applied along the longitudinal axis will inhibit the orientation of the domains along this same axis, giving rise to a favorable domain orientation along the radial and azimuthal axes. This could cause an enhancement of the GMI ratio at low frequencies. However, at high frequencies, the wall mobility is extremely reduced, resulting in a very strong decrease in magnetic permeability [12]. The transverse anisotropy caused by the tensile stress in amorphous wires with vanishing (or slightly negative) magnetostriction reduces the GMI change when the value of the applied stress is large enough. For smaller stresses, the circumferential domain structure of the wires is refined, and the magnitude of the GMI effect can increase [15]. Many works have demonstrated that a commonly observed aspect, resulting from tensile stress, is that the field of the maximum impedance, $H_{peak}$ (which is related to the anisotropy field $H_{k}$), is shifted towards higher values [10,11,12,13,14,15,16,17]. This shift is generally accompanied by a decrease in the value of maximum impedance and consequently of the GMI ratio. In our bending stress experiments, all of these observations were verified.

While the current study deals only with amorphous wires, it could be of great value to perform a similar investigation for the case of nanocrystalline materials in the future. These materials are usually processed with a controlled annealing of an amorphous precursor. Due to their nanometric grain sizes, these alloys exhibit outstanding soft magnetic proprieties, which make them good candidates for the occurrence of the GMI effect. In Fe-rich alloys, nanocrystallization produced by annealing largely enhances the magnitude of the GMI effect [24]. The same behavior as in amorphous alloys could appear in nanocrystalline alloys with an applied tensile stress, provided that they have the same sign of magnetostriction constant [25]. The tensile stress would enhance the transverse anisotropy of the material. The effect could be larger in amplitude, as the transverse anisotropy induced by tensile stress is larger in nanocrystalline alloys than in amorphous alloys [26]. Nevertheless, nanocrystalline materials are less adapted than their amorphous counterparts for our sensing application, as they exhibit poor mechanical properties.

The behavior of the GMI amorphous wires under compressive stress is, to our knowledge, not frequently investigated, despite a very recent experimental study in ribbons [27]. One might, however, argue that, as the magnetostriction constant is negative, a compressive stress applied along the longitudinal axis of the wire will induce a favorable orientation of the domains along this axis, reducing consequently the GMI effect, as the anisotropy is no more circumferential. More quantitative analysis of the wire’s behavior under compressive stress is still, however, necessary.

The effect of bending or flexion stress on the GMI is even less known than the tensile and compressive stresses. In our results, and for the used length and bending curvature of the wire, it seems that this effect gives similar results to those of tensile stress. This observation should be considered cautiously, since the observed effect could be actually dependent on the bending curvature and on the parameters (frequency and amplitude) of the HF excitation current $i_{ac}$. According to this curvature, some competition could exist between tensile and compressive stress.

### 3.2. Sensitivity, Offset, and Reversibility

The change of the GMI ratio and the anisotropy under a bending stress is important to allow for a fundamental and rigorous understanding of the involved physical phenomena. However, the change in the local sensitivity at a given bias field, the change of the offset, and the reversibility are, from a practical point of view, crucial criteria in a real implementation of a GMI sensor involving bending. The quantification of these parameters is therefore essential.

**Figure 5:**
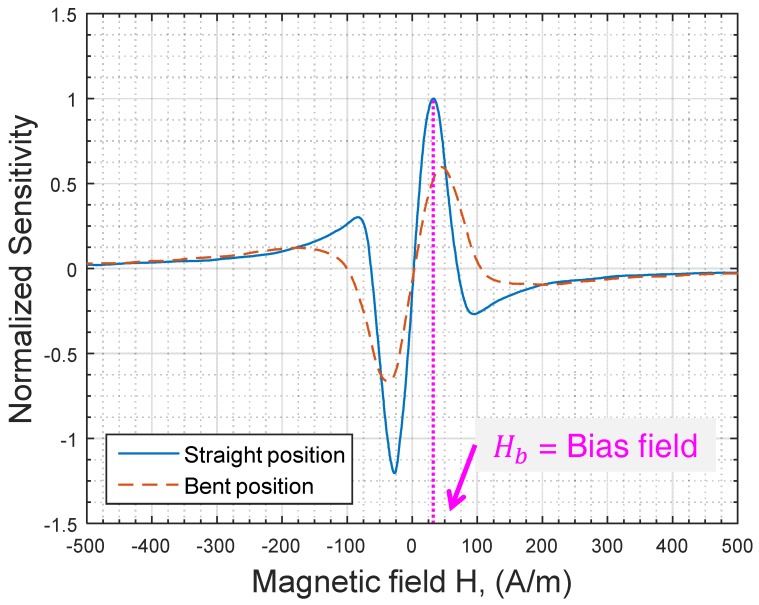
Change of the normalized sensitivity $\frac{1}{S\left(H_{b}\right)}\frac{\partial \left|Z\left(H\right)\right|}{\partial H}$, under bending stress. The sensitivity was normalized with respect to the maximum sensitivity, $S\left(H_{b}\right)$ , obtained in a straight position of the wire.

In the absence of a bending stress, the maximum sensitivity for the used sample was achieved at a field of about 32 A/m (remember that we consider, and without loss of generality, the positive fields region only). In a sensor realization, this is generally chosen as a bias field ($H_{b}$ in Figure 3 and Figure 5). The associated sensitivity is noted $S\left(H_{b}\right)$.

In our experiments, the sensitivity $S\left(H\right)=\frac{\partial \left|Z\left(H\right)\right|}{\partial H}$ was obtained by differentiation of the |Z(H)| curve of Figure 3. For simplicity reasons, we consider a normalized sensitivity with respect to the maximum sensitivity obtained in a straight position of the wire at $H_{b}$ (i.e., $S\left(H_{b}\right)$).

This normalized sensitivity is then defined by $\frac{S\left(H\right)}{S\left(H_{b}\right)}=\frac{1}{S\left(H_{b}\right)}\frac{\partial \left|Z\left(H\right)\right|}{\partial H}$ and is plotted in Figure 5 for both straight and bent positions of the wire.

Then, when the wire is bent, the maximum sensitivity (in the region of positive fields) decreases to about 58% of $S\left(H_{b}\right)$. Moreover, this new maximum is obtained at a higher field of about 45 A/m (Figure 5). This obviously means that, for the design of GMI-based sensors, the value of the initially chosen bias field $H_{b}$ (in the straight position of the wire) will not allow a maximum of sensitivity in bent positions. In fact, at this bias field $H_{b}$, the sensitivity in our sample was reduced to about 52% of its initial value.

The reduction of the maximum sensitivity was roughly consistent with the change of the field of maximum impedance, $H_{peak}$, and the value of the impedance at this same field $\left|Z\left(H_{peak}\right)\right|$. In fact, a rough estimation of the maximum sensitivity could be given by $\left|Z\left(H_{peak}\right)\right|/H_{peak}$ [28]. In our experiments, this ratio was decreased in the bent position to about 51% of its value in a straight position. The general trend between the changes of this ratio and the sensitivity is at least consistent.

In the applications of the GMI sensor that involve repetitive bendings, the quantification of the loss of sensitivity is then required. When the sensor is operating in a closed loop, it is important to ensure that the lower sensitivity, obtained in the bent position, will still be “high” so that the final sensor response will be dependent only on the parameters of the feedback (which could be developer-defined) [6].

Another practical issue to be considered is the change of the offset. At the bias field, $H_{b}$, the impedance $\left|Z\left(H_{b}\right)\right|$ was roughly equal to 64 Ω. Under bending, the change of |Z(H)| at this same field was as high as 38% with $\left|Z\left(H_{b}\right)\right|$ = 40 Ω. The instability of the offset is actually a major issue in a practical realization. An efficient offset canceling device has to be incorporated. Solutions could include the use of a difference amplifier [5] or a symmetrical AC bias field [4]. Each of these techniques has limitations that will not be discussed in this paper.

All the experimental results are summarized in Table 1.

While some solutions could be used to reduce the influence of the variations of the offset and to compensate for the loss in sensitivity in the practical utilization of the sensor (utilization that continuously involves bending stress of the sensitive element), one question must be carefully addressed. The reversibility and repeatability of the bending effect is actually a major concern. In the case of poor reversibility and repeatability, the efficiency of solutions that cancel the offset and compensate for the loss of sensitivity will actually be limited. This is why experimental tests of reversibility and repeatability were conducted on the same sample.

**Figure 6:**
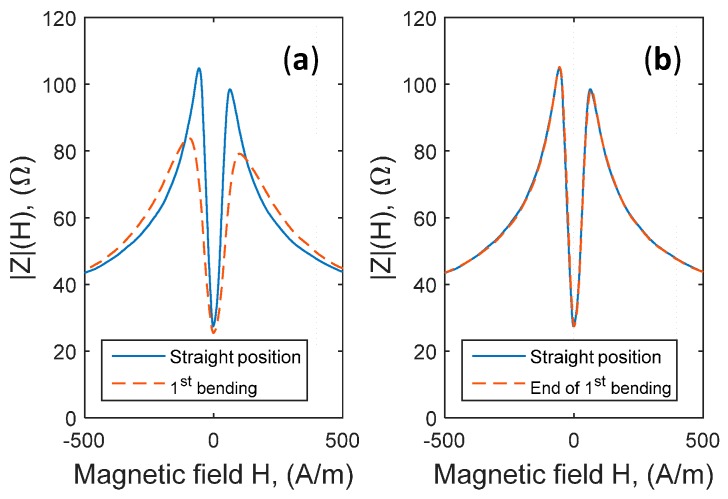

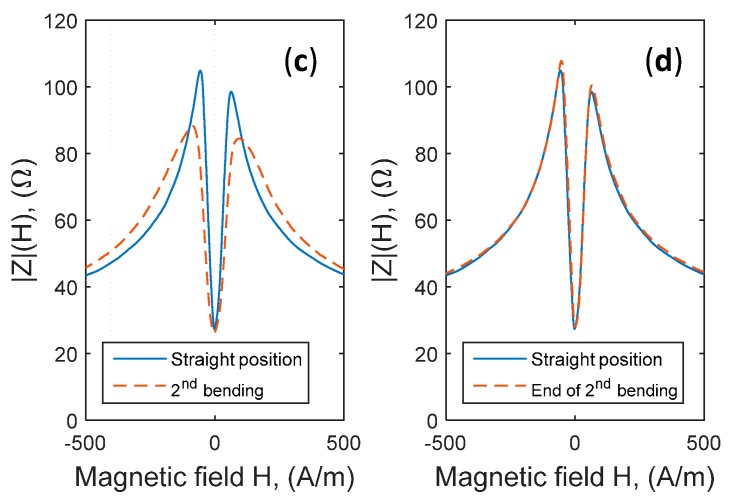
Reversibility and repeatability of the impedance curve, |Z(H)|, following two consecutive bending stresses. (**a**) Change in the modulus of the impedance, |Z(H)| , with a 1st bending stress applied; (**b**) Comparison between the modulus of the impedance after the relaxation of the bending stress (1st bending) and the initial straight position; (**c**) Change in the modulus of the impedance, |Z(H)| , with a 2nd bending stress; (**d**) Comparison between the modulus of the impedance after the relaxation of the bending stress (2nd bending) and the initial straight position.

**Figure 7:**
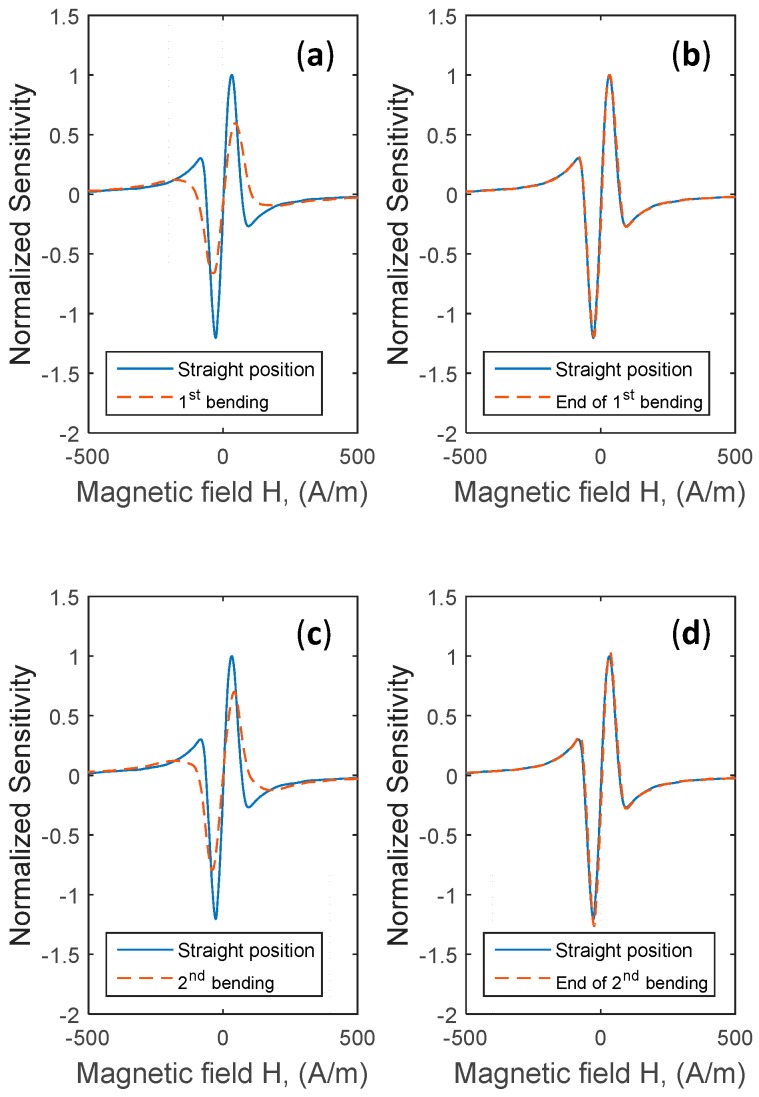
Reversibility and repeatability of the sensitivity following two consecutive bending stresses. (**a**) Change in the normalized sensitivity with a 1st bending stress; (**b**) Comparison between the normalized sensitivity after the relaxation of the bending stress (1st bending) and the initial straight position; (**c**) Change in the normalized sensitivity with a 2nd bending stress; (**d**) Comparison between the normalized sensitivity after the relaxation of the bending stress (2nd bending) and the initial straight position.

The wire was bent under the same conditions described in Section 2 and then relaxed to return to its initial straight position. For example, Figure 6 presents the change in the impedance |Z(H)|. with a first applied stress followed by a relaxation step (a return to the straight position) and then a second applied bending followed by relaxation. Figure 7 shows also the related curves of normalized sensitivities.

We have shown, for illustration purposes only, the effect of two consecutive bendings. About 10 consecutive bendings were conducted and showed that the change in the |Z(H)| curve did not exceed a few percent of the absolute value of |Z(H)|. This value can be included in the error margin of two consecutive measurements (within our current experimental setup, two consecutive applied stresses can slightly differ).

Nevertheless, we can conclude that the first observations showed that the effect of the bending stress was roughly reversible and repetitive (obviously within the limit of elasticity of the wire). This result is actually of great value for sensor applications.

While this study has addressed an initial overall behavior of amorphous GMI Co-rich wires under bending stress, it is still obviously required that these experimental results be confirmed by theoretical analysis of model and completed by further experimental investigations. First of all, and unlike the effect of tensile stress, which has been largely studied in the literature, the effect of compressive stress should be carefully addressed from theoretical and experimental points of view. The general approximation made in considering that the bending effect is a combination of tensile and compressive effects must be refined. One of the two effects could dominate according to the diameter of the curvature of the bent wire. The influence of the frequency and of the amplitude of high frequency excitation current, $i_{ac}$, should also be considered.

All of these issues comprise the subject of our ongoing and future works.

## 4. Conclusions

We addressed an experimental study to evaluate the impact of the bending stress on the GMI effect in amorphous Co-rich wires. This bending stress was considered an influential parameter in our application of electrical current GMI sensors. Its effect on the relevant quantities for GMI sensor implementation was investigated. The results show that the GMI ratio, the intrinsic sensitivity, and the offset in the studied sample were affected by the bending. For the considered experimental conditions of bending and the used parameters of excitation current of the wire, both GMI ratio and maximum sensitivity were reduced (by roughly 50%). A net change in the offset was also observed. It has also been shown that the effect of bending seemed to be reversible and repetitive. However, more intensive work is still necessary to allow for a better understanding of the physical phenomena related to bending and compression. This understanding must also be confirmed by more experimental investigations.

## Figures and Tables

**Table 1:** Summary of the results obtained with an amorphous Co-rich wire, with a bending stress applied.

Physical Quantities	With No Bending Stress Applied	With a Bending Stress Applied (Maximum Strain Applied along the Wire: 800 MPa)	Relative Change (%)
Maximum GMI ratio, ${\left(∆Z/Z\right)}_{max}$	220%	160%	−27%
Peak field, $H_{peak}$ ($H_{peak}\approx H_{k}$)	64 A/m	100 A/m	+56%
Maximum normalized sensitivity, $\frac{1}{S\left(H_{b}\right)}{\frac{\partial \left|Z\left(H\right)\right|}{\partial H}}_{max}$	1	0.58	−42%
Normalized sensitivity at $H_{b}$ (bias field), $\frac{1}{S\left(H_{b}\right)}\frac{\partial \left|Z\left(H\right)\right|}{\partial H}\left(H_{b}\right)$	1	0.52	−48%
Offset, $\left|Z\left(H_{b}\right)\right|$	64 Ω	40 Ω	−38%
